# Metadata retrieval from sequence databases with *ffq*

**DOI:** 10.1093/bioinformatics/btac667

**Published:** 2023-01-05

**Authors:** Ángel Gálvez-Merchán, Kyung Hoi (Joseph) Min, Lior Pachter, A Sina Booeshaghi

**Affiliations:** Division of Biology and Biological Engineering, California Institute of Technology, Pasadena, CA 91125, USA; Department of Computer Science and Electrical Engineering, Massachusetts Institute of Technology, Cambridge, MA 91125, USA; Division of Biology and Biological Engineering, California Institute of Technology, Pasadena, CA 91125, USA; Department of Computing and Mathematical Sciences, Pasadena, CA 91125, USA; Department of Mechanical Engineering, California Institute of Technology, Pasadena, CA 91125, USA

## Abstract

**Motivation:**

Several genomic databases host data and metadata for an ever-growing collection of sequence datasets. While these databases have a shared hierarchical structure, there are no tools specifically designed to leverage it for metadata extraction.

**Results:**

We present a command-line tool, called *ffq*, for querying user-generated data and metadata from sequence databases. Given an accession or a paper’s DOI, *ffq* efficiently fetches metadata and links to raw data in JSON format. *ffq*’s modularity and simplicity make it extensible to any genomic database exposing its data for programmatic access.

**Availability and implementation:**

*ffq* is free and open source, and the code can be found here: https://github.com/pachterlab/ffq.

## 1 Introduction

The extraordinarily large volume of user-generated sequencing data available in public databases is increasingly being utilized in research projects alongside novel experiments (Hippen and Greene, 2021; Huang *et al.*, 2021; Kasmanas *et al.*, 2021; Klie *et al.*, 2021; Lung *et al.*, 2020; Rajesh *et al.*, 2021; Razmara *et al.*, 2019; Simon *et al.*, 2018; Booeshaghi *et al.*, 2022; Wartmann *et al.*, 2021). Collation of metadata is crucial for the effective use of publicly available data. Accurate metadata can provide information about the samples assayed and can facilitate the acquisition of raw data. For example, *sra-tools* enable users to query and download data from the National Center for Biotechnology Information Sequence Read Archive (NCBI SRA), which currently hosts 13.67 PB of data. An alternative to *sra-tools* is the *pysradb* tool (Choudhary, 2019). *pysradb* was developed to access metadata from the SRA, using metadata obtained from the regularly updated SRAdb SQLite database (Zhu *et al.*, 2013). MetaSRA adds additional standardized metadata on top of the SRAdb SQLite database (Bernstein *et al.*, 2017) and also provides an application programming interface (API) for accessing them. While these and other tools (Bernstein *et al.*, 2020; Eaton, 2020; Li *et al.*, 2018; Mahi *et al.*, 2019) have proven to be very useful, they provide access to a limited scope of databases. We developed *ffq* to facilitate metadata retrieval from a diverse set of databases, including

1. NCBI SRA and Gene Expression Omnibus (GEO),
2. European Molecular Biology Lab-European Bioinformatics Institute European Nucleotide Archive (EMBL-EBI ENA),
3. DNA Data Bank of Japan Gene Expression Archive (DDBJ GEA) and
4. Encyclopedia of DNA Elements (ENCODE) database (Davis *et al.*, 2018; ENCODE Project Consortium, 2012).

**Fig. 1.**
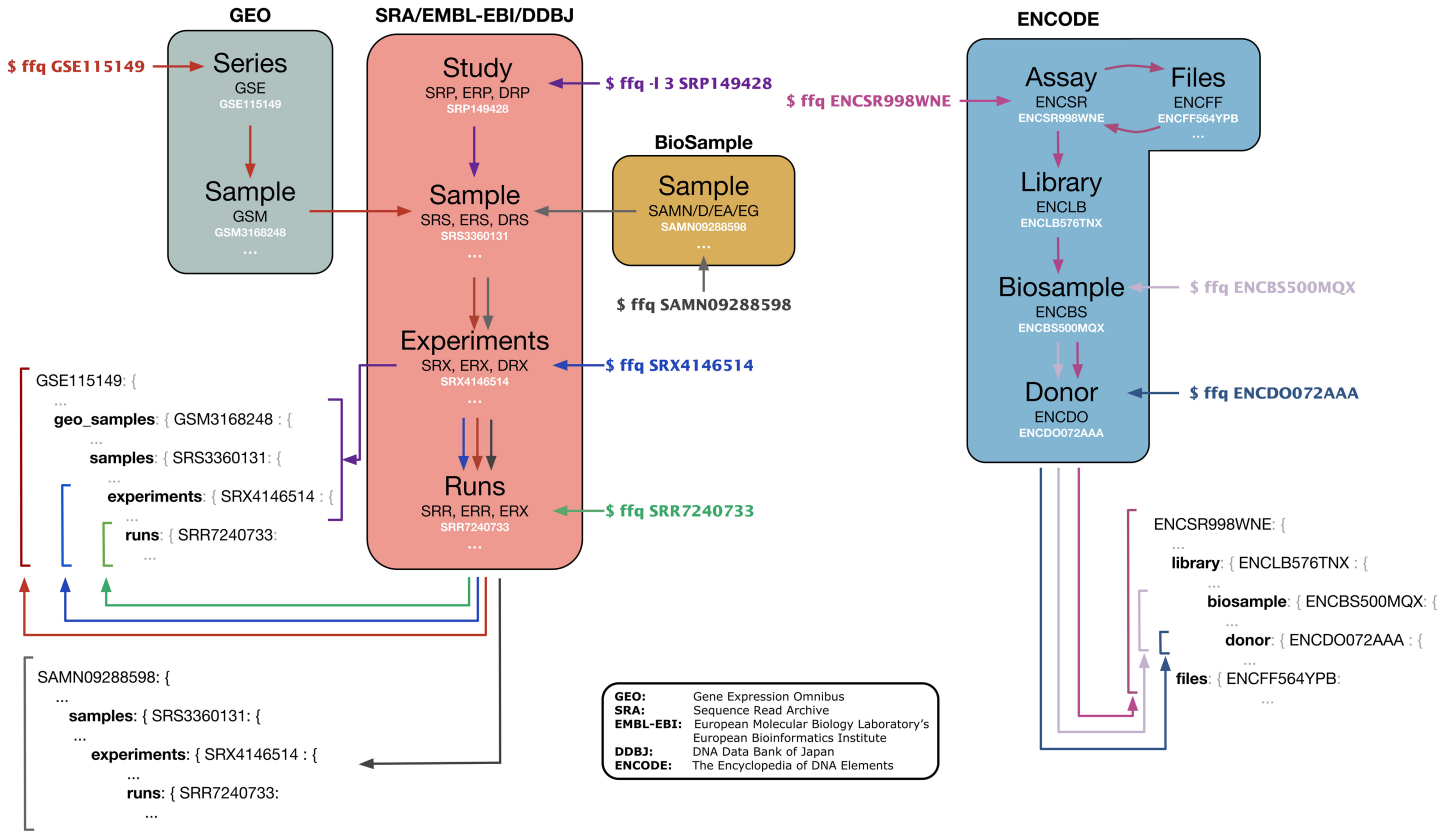
Metadata retrieval. *ffq* fetches and returns metadata as a JSON object by traversing the database hierarchy. Subsets of the database hierarchy can be returned by specifying *-l [level]*

In order to facilitate a modular architecture for *ffq*, we first studied the structure of these databases in detail to identify commonalities and relationships between them (Fig. 1).

The SRA, ENA and DDBJ databases all follow a similar hierarchical structure where studies are grouped into samples, experiments and runs, a shared architecture that is useful and likely the result of the longstanding International Nucleotide Sequence Database Collaboration (INSDC) between the ENA, NCBI and DDBJ. We note that the Genome Sequence Archive (Chen *et al.*, 2021; CNCB-NGDC Members and Partners, 2022) is not a member of the INSDC. However, it also uses a similar hierarchical structure for its database and regularly ingests data from the SRA but does not expose its publicly available data for programmatic access.

The consistent database schemas used by members of the INSDC greatly simplify metadata retrieval for *ffq*. For example, GEO accession codes are grouped hierarchically through Series and Samples and have external relations to SRA accession codes for raw sequencing data submitted to the SRA. This enables *ffq* to fetch metadata and processed data from GEO that submitters have associated with raw sequencing data stored in the SRA.

## 2 Description

Based on the database architectures, we created *ffq* to fetch metadata using database accessions or paper DOIs as input. Importantly, *ffq* only fetches metadata and links to data files and does not offer data downloading. This deliberate design decision was motivated by the UNIX philosophy ‘Make each program do one thing well’ (McIlroy *et al.*, 1978).

The *ffq* options are summarized below:

- *ffq* [accession(s)]
  - Where [accession] can be any of the following: SR(R/X/S/P), ER(R/X/S/P), DR(R/X/S/P), GS(E/M), ENC(SR/BS/DO), CXR, SAM(N/D/EA/EG) and DOI.
- *ffq* [-l level] [accession(s)]
  - Where [level] defines the hierarchy in the database to which data is subset data.
- *ffq* [–ftp] [–aws] [–gcp] [–ncbi] [accession(s)]
  - Where the flags correspond to the types of data-storage links for the raw data.
- *ffq* [-o out] [–split] [accession(s)]
  - Where [out] corresponds to a path on disk to save the JSON file and [–split] splits the metadata from multiple accessions into their own file.

The *ffq* codebase consists of 58 functions and 2198 lines of code across six files and relies on only four software dependencies. Users supply an accession or DOI and the tool returns metadata for the sequencing data associated with that accession or DOI.

Accession-based *ffq* metadata retrieval uses the NCBI Entrez programming utilities, ENA API, GEO FTP and ENCODE API to programmatically access metadata with HTTP requests. DOI-based metadata retrieval first converts the DOI to the manuscript title via the CrossRef API (Hendricks *et al.*, 2020) and then retrieves all study accessions associated with the manuscript title with the ENA search API. The reliance on these external dependencies can make it challenging to track API updates that may break *ffq* functionality. To provide resilience to such changes, we have implemented extensive quality control via an automated testing framework that validates behavior against all external APIs and five Python versions (3.6, 3.7, 3.8, 3.9 and 3.10) that cover 78% of the code. This makes it easy to detect and address API updates within *ffq*.

Once fetched, metadata is returned as a Javascript Object Notation (JSON) object. Run times for metadata retrieval vary depending on database up-time, server connection speed and database rate-limiting, but generally, we find that *ffq* can download metadata at a rate of 10 s per sample. This rate includes short and deliberate delays we have added between HTTP requests to prevent a perceived Denial-of-Service.

External factors may impact *ffq’*s ability to fetch metadata that are independent of the tool. Internet connection, improperly formatted accessions, missing or incomplete metadata are some of the failure modes that users may face. To aid users in debugging missing or incomplete metadata, custom exceptions have been implemented and possible failure modes and caveats have been listed in the documentation.

## 3 Usage and documentation

The *ffq* tool is written in Python and can be installed with pip and conda. Users supply an accession or DOI and the tool returns metadata for the associated sequencing data. The JSON-return objects make *ffq* interoperable with other tools such as *jq* for easy command-line parsing. Additionally, *ffq*’s modularity and simplicity make it extensible to other genomic databases. By leveraging existing APIs, *ffq* offers a lightweight solution for querying data that is guaranteed to be more up-to-date than tools that rely on regular database builds.

**Fig. 2.**
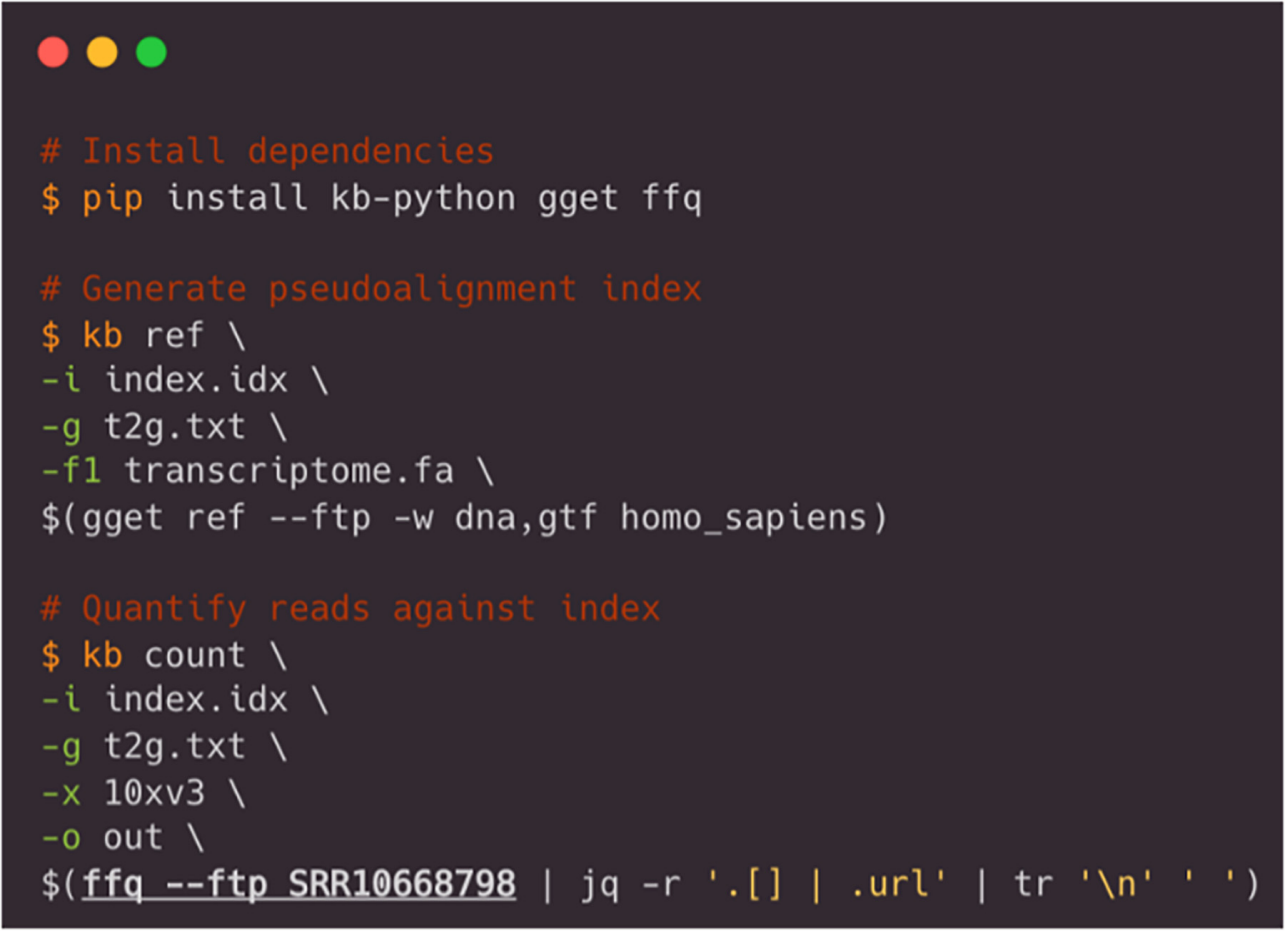
Example use case. Publicly available scRNAseq data are fetched with *ffq* and quantified with *kb-python* to generate a gene count matrix. The *ffq* command is underlined

These features enable researchers to use *ffq* to refine research questions. For example, *ffq* can be used to fetch publicly available scRNAseq data, which can be preprocessed with existing tools (Melsted, *et al.*, 2021) and compared against newly generated data (Fig. 2). Alternatively, *ffq* can be used for sequencing quality control; sequencing reads can be fetched with *ffq* and piped into common command-line tools to count the number of reads or assess the per-base quality scores. These and other use cases are explained in the *ffq* documentation. The modularity of *ffq* makes possible streamwise processing of publicly available FASTQ files for any number of applications.

## 4 Discussion

While *ffq* facilitates downloading of data from numerous genomic databases, the results retrieved are only useful to the extent that the metadata uploaded is meaningful and complete. Meaningful and complete user-generated data underlies the curation of genomic references essential for comparative genomic data analysis (Luebbert and Pachter, 2023). Unfortunately, there is little to no standardization of user-uploaded sequencing metadata (Rajesh *et al.*, 2021; Wang *et al.*, 2019), and metadata descriptions can become exceedingly complex for current multiplexed experiments, where different assays with distinct data types are combined. Improvement of metadata uploading in machine-readable standard formats is essential if publicly available genomic data are to be usable by scientists in the future.

Users who wish to refine research questions with complete and accurate publicly available data will benefit from *ffq*. By providing direct links to sequencing data and metadata, *ffq* allows any number of downstream procedures that operate on sequencing reads. Importantly, the modularity of *ffq* enables streamwise processing of data and metadata that obviates the need for large amounts of storage and lessens the cost of computing.

## Data Availability

All data and code associated with this manuscript is available at https://github.com/pachterlab/ffq.
